# Dissecting HIV's Latent Menace

**DOI:** 10.1371/journal.pbio.1001209

**Published:** 2011-11-29

**Authors:** Caitlin Sedwick

**Affiliations:** Freelance Science Writer, San Diego, California, United States of America

**Figure pbio-1001209-g001:**
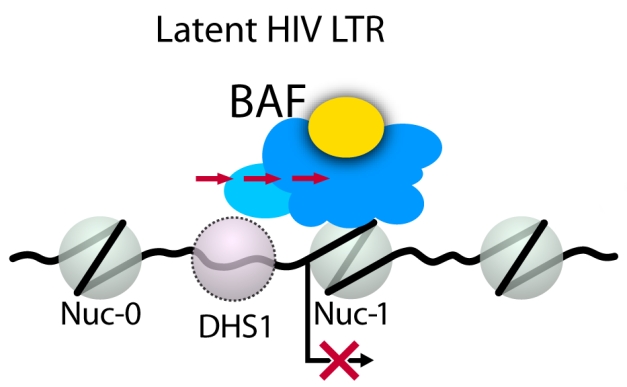
BAF actively represses latent HIV by pushing/pulling the nuc1 nucleosome from its energetically preferred location (DHS1) to a location that obscures the HIV transcriptional start site.

[Fig pbio-1001209-g001]When HIV infects a T lymphocyte, it first inserts a copy of its genome into the cell's DNA. This inserted virus, called a provirus, then races to make as many new viruses as possible before its host cell dies. But in a few infected cells, HIV does not immediately turn its host into a viral factory. Instead, the provirus is carried around in the DNA of the cell as a transcriptionally silent—or latent—passenger, only to explode back into action at a later time, when its host cell attempts to participate in an immune response to infection by other pathogens.

Because they target the products of HIV transcription, current antiviral therapies like HAART can't kill latent HIV. And because a full-blown infection can be re-established from a tiny reservoir of latently infected cells, viral latency is an important contributor to our struggle against HIV. In a paper published this month in *PLoS Biology*, Haleh Rafati, Tokameh Mahmoudi, and colleagues provide new insights into how HIV establishes latency.

Because viruses frequently hijack cellular processes for their own purposes, it is often instructive to examine how that co-option takes place. For example, it's known that cells can prevent transcription of entire stretches of their DNA by modifying the ability of the cellular transcriptional machinery to access that DNA.

Most DNA is packed into structures called nucleosomes, which consist of DNA wrapped around a protein complex called a histone; nucleosomes, strung together by short stretches of DNA, give DNA the appearance of beads on a string. Certain DNA sequences are more energetically favorable for the formation of nucelosomes than are others. But sometimes the mere presence of a nucleosome structure spanning a particular stretch of DNA reduces gene transcription by obscuring the DNA sequences upon which the transcriptional machinery is assembled. However, nucleosomes can be repositioned with the help of proteins, including those of the SWI/SNF family.

SWI/SNF is actually a complex of proteins assembled from several subunits. There are two main varieties of SWI/SNF present in mammalian cells: one, called BAF, uniquely contains a subunit named BAF250, while the other, called PBAF, contains instead a different subunit. Earlier work conducted in Mahmoudi's and others' labs had produced conflicting results on how SWI/SNF proteins affect transcription of the HIV provirus—in some cases, SWI/SNF seemed to promote viral transcription and in others to inhibit it. Therefore, Rafati and colleagues decided to take a closer look at how SWI/SNF affects HIV transcription.

To this end, the researchers used siRNA to deplete a protein subunit common to all SWI/SNF varieties in cells harboring latent HIV provirus. They found that this treatment increased the virus' baseline transcription rates, suggesting that SWI/SNF promotes HIV latency. Follow-up experiments using siRNA depletion of BAF- or PBAF-specific subunits, however, showed that only BAF contributes to latency.

How does BAF promote HIV latency? Previous studies had shown that there are two nucleosomes present on the portion of the HIV provirus where the cellular transcription machinery is assembled, preparatory to viral transcription. Rafati and colleagues found that BAF repositions one of these nucleosomes in such a manner it blocks viral transcription. Furthermore, they showed that the site where BAF positions this nucleosome is actually less well favored for nucleosome formation than a neighboring region that, in the presence of BAF, does not contain any nucleosome. Therefore, BAF is actively working to position a nucleosome at this site, thereby preventing HIV transcription.

But even though BAF acts to promote viral latency, HIV is still able to emerge from this repressed state if presented with the right circumstances. For example, as mentioned earlier, latent HIV remains dormant in its host cell until that cell is called upon to participate in an immune response. Rafati and colleagues wanted to see what happens when HIV emerges from latency, so they used a chemical treatment to mimic the mobilization of latently infected cells to an immune response. Under these conditions, they observed, the BAF-positioned nucleosome disappears from its inhibitory placement on the provirus. BAF itself is also dislodged from its association with the proviral DNA, and is replaced by PBAF. The swapping of BAF for PBAF on proviral DNA appears to be important, the researchers found, because PBAF then helps promote maximal HIV transcription.

The finding that different SWI/SNF complexes are required at different times in HIV's life cycle has several interesting implications. One of these is reflected by the authors' observation that depletion of BAF reduces the ability of HIV to establish latency in the first place. As pointed out by Rafati et al., these findings make BAF a tantalizing therapeutic target in the ongoing battle against HIV.

**Rafati H, Parra M, Hakre S, Moshkin Y, Verdin W, et al. (2011) Repressive LTR Nucleosome Positioning by the BAF Complex Is Required for HIV Latency. doi:10.1371/journal.pbio.1001206**

